# Mr. Samuel at the Poor-Law Conference

**Published:** 1915-02-13

**Authors:** 


### MR. SAMUEL AT THE POOR-LAW CONFERENCE.

The brief and eloquent speech with which Mr. Herbert Samuel, the President of the Local Government Board, opened the proceedings of the Central Poor-Law Conference on Tuesday last was concerned chiefly with a short *résumé* of the problems due to the war with which the Poor Law has had to deal, and of the methods by which the difficulties have been met.

The one reform to which Mr. Samuel committed himself and his Department was in connection with the relief of women and children. He said that no Board of Guardians could be considered to be carrying out its duties efficiently unless it had on its staff a lady visitor. He admitted that the same principle ought to be applied to the central authority, and he stated that the Local Government Board were taking steps to provide a woman inspector for every general inspector's district. Pursuing the same line of argument, he paid a high tribute to the excellent work performed by lady Guardians, and expressed the opinion that every Board of Guardians ought to possess a percentage of women amongst its members. These views will meet with general approval. There are many obvious instances in which the work of the Guardians can only be satisfactorily initiated and carried out through women Guardians and women officials.

Turning to the special problems which had arisen or were likely to arise as a result of the war, Mr. Samuel said that the central authority had to acknowledge with gratitude that in every step it had taken or had suggested it had met with the greatest assistance from Boards of Guardians. At the outset of the war there had been grave fears of a widespread dislocation of trade and consequent increase of unemployment. But fortunately the problem had really resolved itself into a transference of labour from those trades which had been diminished by the war to those which had been stimulated by it. Distress actually due to the war, the Local Government Board felt, so far as possible, ought to be met, not by public funds, but by charitable donations. The result had been far greater than anyone could have hoped. Including the Colonial contributions, more than £5,000,000 had been provided by the public. It was felt that no existing body could satisfactorily administer these funds, and special bodies had therefore been called into being. Upon these special bodies the Local Government Board realised that the Guardians were pre-eminently entitled to representation. It had pointed this out to the County Councils, and he was glad to know that in every district throughout Great Britain the Guardians had received the recognition which was their due. The great bulk of the fund—more than four-fifths—was still untouched, and would be available to alleviate the distress which was bound to arise when the war was finished.

Thus to those who expected that Mr. Samuel would take advantage of the occasion to propound his views as to the future of Boards of Guardians or to indicate the reforms he considers urgent and desirable, the speech will prove a disappointment. A little consideration will show the unreasonableness of such a feeling. As Mr. Samuel said, at a time when the whole intellectual, physical, and moral capacity of the nation is straining towards the achievement of a single purpose—the defeat of a powerful, implacable, and ruthless enemy—domestic reforms and great legislative changes must remain in abeyance. The reasonableness of the statement cannot be gainsaid, and from it it is surely justifiable to derive this consoling corollary, that the statement would not have been made if Mr. Samuel had not felt that under normal conditions the time was ripe for a comprehensive scheme of Poor-Law reform.

